# Yield of systematic household contact investigation for tuberculosis in a high-burden metropolitan district of South Africa

**DOI:** 10.1186/s12889-019-7194-2

**Published:** 2019-07-03

**Authors:** N. Gladys Kigozi, J. Christo Heunis, Michelle C. Engelbrecht

**Affiliations:** Centre for Health Systems Research & Development, University of the Free State, P.O. Box 399, Bloemfontein, 9300 South Africa

**Keywords:** Active case finding, Free State Province, Index cases, Household contact investigation, Mangaung Metropolitan District, Tuberculosis

## Abstract

**Background:**

Systematic household contact investigation (SHCI) is recommended as an active-case-finding (ACF) strategy to identify individuals at high risk of tuberculosis (TB) infection, in order to enable early detection and treatment. Reluctance to implement SHCI in sub-Saharan African and South African high-burden contexts may stem from uncertainty about the potential yield of this strategy when targeting specific categories of TB index cases. In order to inform and motivate scale-up, this pilot study investigated the effectiveness of SHCI when targeting the World Health Organization’s (WHO) recommended categories of infectious index cases.

**Method:**

Data were gathered in September and October 2016. Household contacts of infectious TB cases who attended 40 primary health care facilities in Mangaung Metropolitan District were recruited. The categories of TB index cases included 1) children <5 years, 2) HIV co-infected pulmonary TB (PTB) cases (≥5 years), 3) HIV-negative PTB cases (≥5 years), and 4) multidrug-resistant (MDR) TB cases. Contacts were screened for TB symptoms and symptomatic individuals and all children <5 years were referred for clinical evaluation. Data were analysed to establish the yield and factors associated with new TB diagnosis.

**Results:**

Of 259 contacts screened, just under half (47.1%) underwent TB clinical investigation, during which 17 (6.6%) new TB cases were diagnosed, which represents a prevalence rate of 6564 per 100,000 population. Fifteen contacts needed to be screened to detect one new TB case. The proportion of new TB cases was the highest among contacts of HIV-negative PTB index cases (47.9%). The likelihood of TB diagnosis was higher among male contacts (odds ratio [OR]: 4.8; 95% confidence interval [CI]: 1.54–14.97) and those reporting coughing (OR: 4.3; 95% CI: 1.11–16.43).

**Conclusion:**

The high yield of new TB observed in this pilot study demonstrates that targeted SHCI may be an effective ACF strategy in Mangaung and similar high-burden settings in South Africa. Targeting different index case categories produced variable yield – the highest among contacts of HIV-negative TB index cases. SHCI among household contacts of all four the WHO-recommended categories of infectious TB index cases – and male and coughing contacts, in particular – should be maximised.

## Background

In 2016, South Africa recorded the highest TB incidence and mortality rates among the 30 high-burden countries, at respectively 781 and 222 per 100,000 population [1]. This pilot study was conducted in the Free State Province, where, in 2016, the TB mortality rate, at 69.7 per 100,000 population, was substantially higher than the national rate of 52.8 per 100,000 population [2]. More specifically, the research took place in Mangaung Metropolitan District, which, in 2016, had a population of 787,803 [3]. In 2015, the TB incidence rate in Mangaung was higher than the national rate, at 616 and 520 per 100,000 population respectively. In 2014, the estimated TB-related death rate in the district, at 9.7%, was substantially higher than the national rate of 6.1% [4]. The high TB burden and poor treatment outcomes informed the choice of this province and district for the study.

The World Health Organization (WHO) recommendations for investigating contacts of persons with infectious TB in low- and middle-income countries endorse household contact investigation as one of the active-case-finding (ACF) strategies that can be used to identify individuals at high risk of TB infection in communities [5]. Various recent studies emphasise the importance of household contact investigation [6–9], which enables early TB detection, including identification of latent TB infection, thereby enabling preventive measures and prompt treatment initiation [5, 10].

Although policy makers and researchers worldwide recognise the necessity of more aggressive ACF approaches to supplement passive case finding [10–16], robust evidence for the effectiveness of such approaches in high-burden sub-Saharan African settings is wanting [17, 18]. Indeed, while the WHO classifies household contact investigation as a “strong recommendation”, it concedes that the recommendation is based on “very low-quality evidence” [5], p., 8. At the country level, this may discourage programme-wide implementation of interventions [19].

Systematic screening for active TB involves the systematic identification of people with suspected active TB in a predetermined target group, by using tests, examinations or other procedures that can be applied rapidly [10]. The WHO [5] recommends that contact investigation should be conducted for household and close contacts when the index case has any of the following characteristics: 1) is a child <5 years, 2) has HIV co-infected pulmonary TB (PTB) (≥5 years), 3) has HIV-negative PTB (≥5 years) or 4) has multidrug-resistant (MDR) TB.

Although TB programmes worldwide have adopted ACF, infrequent or inconsistent investigation of TB patient contacts remains a serious challenge [5, 20–22]. This is also experienced nationally in South Africa [18, 23, 24], and in the Free State province, in particular [25]. Reluctance to implement systematic household contact investigation (SHCI) in the African and South African high-burden contexts may stem from uncertainty about the potential yield of this strategy when targeting specific categories of TB index cases. This study investigated the effectiveness and yield of SHCI when targeting the WHO-recommended categories of infectious TB index cases, in order to inform and motivate SHCI scale-up efforts.

## Methods

### Design and setting

This pilot study was conducted among purposively-selected household contacts of infectious TB patients attending services at 40 primary health care (PHC) facilities in the Mangaung Metropolitan District.

### Participants and sampling

The study targeted household contacts of infectious TB patients with bacteriologically confirmed TB, or who had started TB treatment based on clinical presentation, X-ray findings or other tests in line with national TB management guidelines. At each PHC facility, the TB focal person assisted the research team to identify infectious TB index patients. Patients of any age were eligible for recruitment if they were within three months of initiating treatment, reported at least one household contact, had consented to a household visit by the fieldwork team, and had provided a traceable home address. The caretakers of children <18 years were approached to provide consent. Index cases who were exclusively on extra-pulmonary TB treatment, those who had been on treatment for longer than three months, those who indicated having no household contacts, and those who did not consent to a home visit, were excluded from the study. The WHO-recommended infectious index case categories were purposefully selected at each PHC facility. Efforts were made to recruit at least one index case from each index case category.

Selection of household contacts was also purposive. Household contacts of any age were included in the study if they were not receiving TB treatment at the time of the home visit, had spent at least three months under the same roof as the infectious TB index case, had consented to the study, and were available for interviews during the household visits.

### Household visits and data gathering

**Fig. 1:**
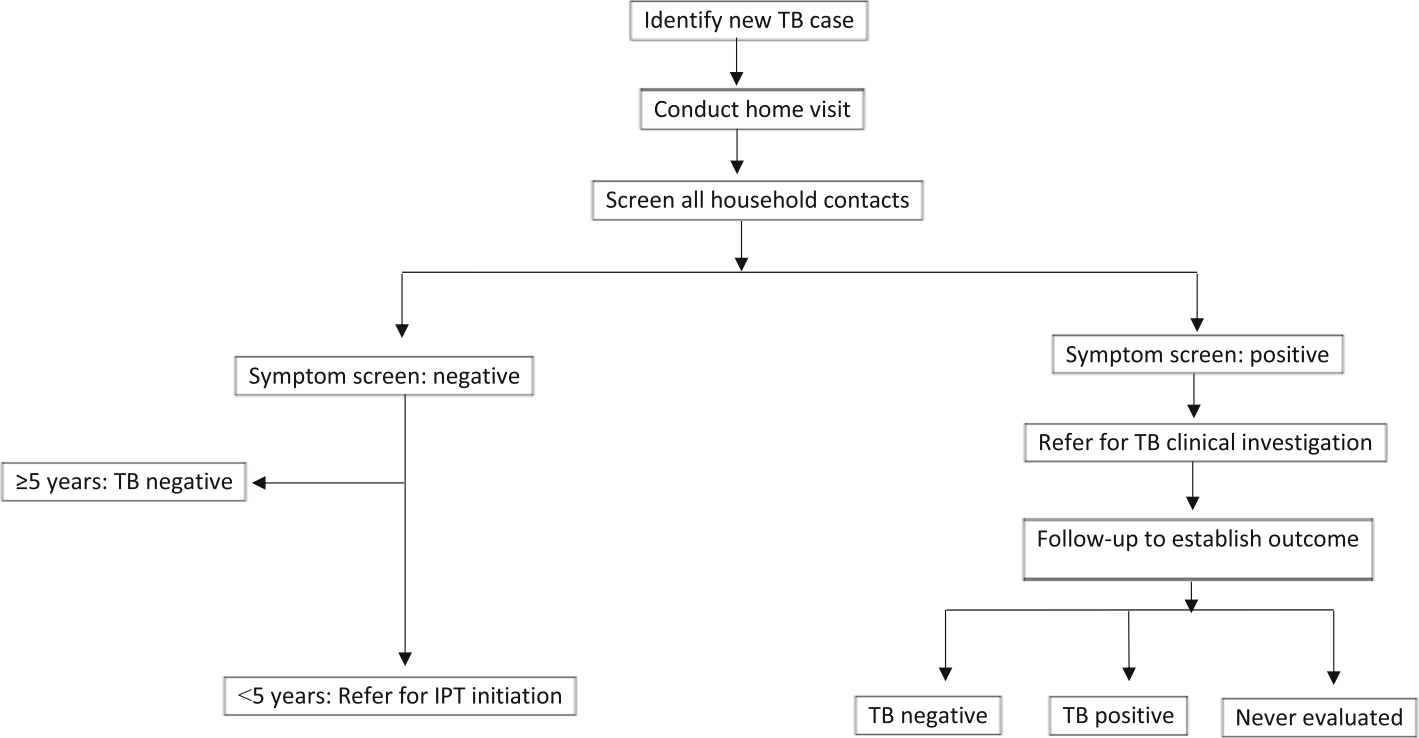
Procedure. IPT = Isoniazid preventive therapy

Data were gathered between 1 September and 31 October 2016. Figure 1 depicts the process followed during data gathering. Consent procedures were administered to the index cases before they were asked to provide contact details of all members of their household. Fieldworkers sought permission to visit the index cases’ households to screen their contacts for TB symptoms, and also to extract clinical information from the index cases’ medical records at the PHC facilities.

Household visits were conducted within one week of index case recruitment and all contacts available at the time of the visit were considered for the study. Consent was obtained from the household contacts, after which they were screened for TB symptoms, including coughing for longer than two weeks, weight loss, fever and night sweats. In addition to caretaker consent, assent was obtained for children 6–17 years. Demographic and other clinical information, such as uptake of HIV testing, was also collected. Caretakers were interviewed on behalf of children <18 years.

In line with TB programme guidelines, all children <5 years (regardless of their initial screening results), as well as symptomatic individuals ≥5 years, were referred to the PHC facility for further clinical evaluation. Telephonic follow-up was conducted seven days after the household visits, to determine whether household contacts had actually attended the PHC facility for clinical evaluation and to record the outcome of the evaluation. Where there was non-compliance, fieldworkers made up to two repeat visits to households to encourage the referred contacts to take up clinical investigation.

### Statistical analysis

The data were analysed using SPSS Version 24 and described using frequencies and percentages. The primary outcome was the yield of SHCI, defined as the number of new TB cases identified per household contact screened. The number needed to screen (NNS) in order to identify one new TB case was expressed as the total number of household contacts screened divided by the number of new TB cases. Binary logistic regression was used to examine the association between certain index case and household contact characteristics and new TB diagnosis. Statistical significance was considered at *p* < 0.05 and 95% confidence interval.

## Results

**Fig. 2:**
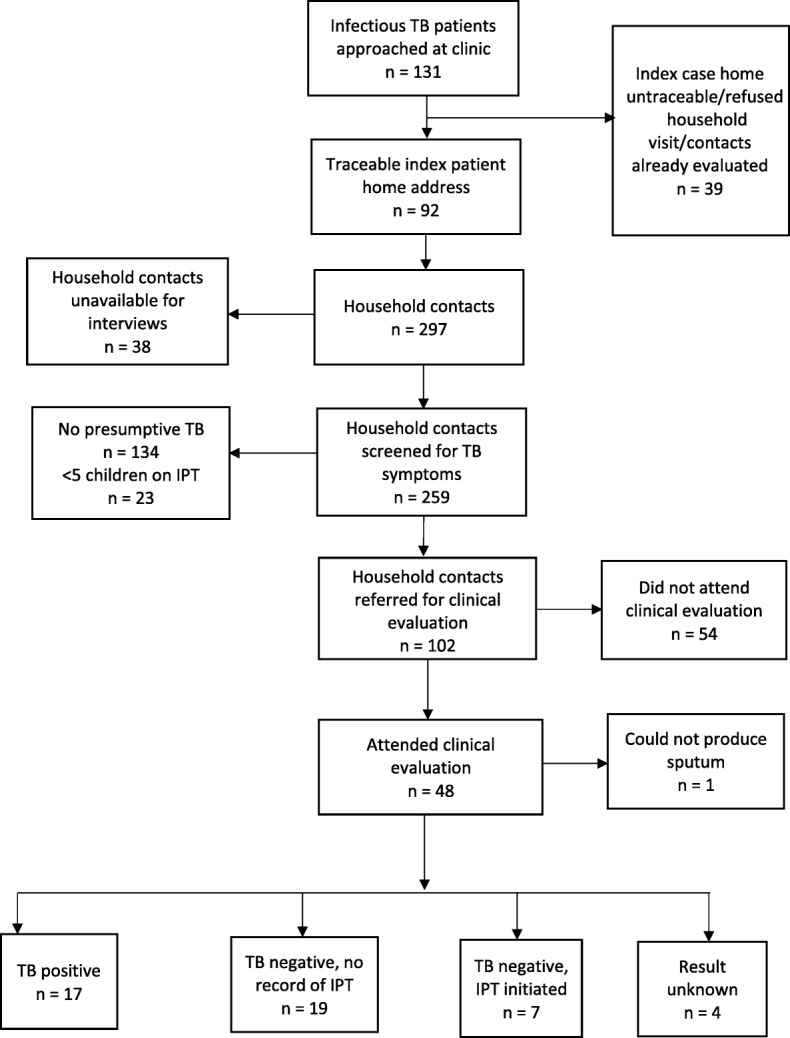
Flow chart of index cases and household contacts included in the study. IPT = Isoniazid preventive therapy

Figure 2 presents the number of infectious TB index cases and household contacts recruited for the study. A total of 131 index cases were approached, of whom 92 (70.2%) provided traceable home addresses and were included into the study. The 92 (70.2%) index cases were linked to a total of 297 household contacts. Out of the 297 household contacts, 259 (87.2%) were available for interviews and were also screened for TB symptoms on the day of the field visit. These individuals were included in the analysis. Of the 38 individuals who were not available for interviews, 10 (26.3%) refused to be interviewed and 28 (73.7%) had conflicting work or travel commitments.

Table 1 depicts the characteristics of the TB index cases (*n* = 92) and the household contacts screened for TB (*n* = 259). Regarding the index cases, just more than two thirds (*n* = 63; 67.0%) were male and one quarter (*n* = 23; 25.0%) were adults aged 35–44 years. Clinical records showed that just more than one quarter (*n* = 24; 26.1%) of these patients had a history of TB diagnosis and almost all (*n* = 90; 97.8%) had known HIV status, of whom half (*n* = 46; 50.0%) were HIV-negative. Further analysis showed that index cases were considerably older than the household contacts: index case median age: 35 years; interquartile range [IQR]: 24–45 years) vs. contact median age: 20 years (IQR: 8–41 years). Most (*n* = 154; 59.5%) household contacts were female. The median number of household contacts for every index case was four (IQR: 3–5).

**Table 1:** Demographic and clinical characteristics of patients and their household contacts

Characteristic	Index cases (*n* = 92)	Contacts TB screened (*n* = 259)	Contacts referred for clinical evaluation (*n* = 102)	Contacts who underwent clinical evaluation (*n* = 48)
	n (%)	n (%)	n (%)	n (%)
Male	63 (68.5)	105 (40.5)	48 (47.1)	26 (54.2)
Age in years^a^				
< 5	12 (13.0)	36 (13.9)	13 (12.7)	8 (16.7)
5–15	3 (3.3)	73 (28.2)	28 (27.5)	15 (31.3)
16–24	8 (8.7)	33 (12.7)	15 (14.7)	6 (12.5)
25–34	22 (23.9)	34 (13.1)	14 (13.7)	5 (10.4)
35–44	23 (25.0)	25 (9.7)	7 (6.9)	2 (4.2)
45–54	13 (14.1)	19 (7.3)	8 (7.8)	4 (8.3)
55–64	7 (7.6)	18 (7.0)	6 (5.9)	3 (6.3)
65+	4 (4.3)	21 (8.1)	11 (10.8)	5 (10.4)
Previously diagnosed with TB	22 (24.2)	33 (12.7)	21 (19.6)	8 (16.7)
Shares bedroom with TB index case		94 (36.3)	52 (44.1)	19 (39.6)
Ever tested for HIV (self-report)	92 (100)	228 (88.0)	101 (82.4)	43 (89.6)
HIV test result^b^				
HIV-positive	44 (47.8)			
HIV-negative	46 (50.0)			
Not recorded	2 (2.2)			

^a^Median age (interquartile range [IQR]) — index case: 35 (24–45) years and household contacts: 20 (8-41) years

^b^TB index case HIV status was verified from medical records at PHC facility; contacts’ HIV status was self-reported and could not be verified

In respect of household contacts, about four in every ten were male (*n* = 105; 40.5%), and the largest proportion (*n* = 73; 28.2%) were aged 5–15 years. About one tenth (*n* = 33; 12.7%) of the contacts had a history of TB diagnosis and just over one third (*n* = 94; 36.3%) shared a bedroom with an infectious index case. A large majority (*n* = 228; 88.0%) of the contacts self-reported having tested for HIV. However, due to the confidential nature of results, most contacts refused to disclose their HIV status and, for those who did, the results could not be verified and are, therefore, not reported. While two in every five (*n* = 102; 39.4%) household contacts were referred for clinical evaluation after symptom screening, more than half (*n* = 54; 52.9%) did not take up this evaluation (Table 1).

Table 2 indicates the yield of SHCI. From the systematic household symptom screening, referral and clinical evaluation exercise, 17 new TB cases were identified. This represents an overall yield of 6.6% and prevalence rate of 6564 per 100,000 population. The yield ranged from nil among contacts of MDR TB patients (no XDR-TB index cases were identified) to 8.0% among contacts of HIV-negative (≥5 years) index cases. For all contacts, the NNS to diagnose one new TB case was 15. The NNS ranged from 13 among contacts of HIV-negative index cases, to 18 among contacts of HIV-positive patients.

**Table 2:** Yield of systematic household contact investigation

Variable	Household contacts screened n	Number of new TB cases n	Yield %	NNS
All participants	259	17	6.6	15
Index case category				
Children < 5 years	29	2	6.9	14
HIV-positive (≥5 years)	124	7	5.6	18
HIV-negative (≥5 years)	100	8	8.0	13
MDR-TB (≥5 years)	6	0	0	^a^

^a^Only one contact was symptomatic, clinical evaluation did not yield new TB

*NNS* number needed to screen

The majority of the 17 new TB cases (*n* = 12; 70.6%) were male. Male gender was independently statistically significantly (OR: 3.9; CI: 1.31–11.37) associated with the likelihood of a laboratory-confirmed TB diagnosis. After controlling for other variables in the model, male contacts were 4.8 (CI: 1.54–14.97) times more likely to be diagnosed with TB than their female counterparts. Household contacts who self-reported coughing were 4.3 (CI: 1.11–16.43) times more likely to have been diagnosed with TB than those without a cough (Table 3).

**Table 3:** Factors associated with likelihood for new TB diagnosis among household contacts

Variable	Diagnosed with TB (*n* = 17) n (%)	Unadjusted OR (95% CI)	Adjusted OR (95% CI)
Sex			
Female (ref)	5 (29.4)	1	1
Male	12 (70.6)	3.9 (1.31–11.37)	4.8 (1.54–14.97)
Age (median; IQR)	18 (7–43)	1.0 (0.97–1.02)	1.0 (0.97–1.01)
Number of household contacts from same household (median; IQR)	4 (2–5)	1.0 (0.76–1.31)	1.0 (0.76–1.32)
Whether index case is coughing			
No (ref)	5 (29.4)	1	1
Yes	12 (70.6)	2.2 (0.74–6.36)	2.8 (0.84–9.05)
Index case HIV status			
Negative (ref)	10 (58.8)	1	1
Positive	7 (41.2)	0.5 (0.18–1.32)	0.6 (0.19–1.60)
Whether contact shares bedroom with index case			
No (ref)	8 (47.1)	1	1
Yes	9 (52.9)	2.2 (0.81–6.10)	2.3 (0.78–6.55)
Whether household contact is coughing			
No (ref)	13 (76.5)	1	1
Yes	4 (23.5)	2.9 (0.88–9.73)	4.3 (1.11–16.43)

*Ref* reference, *IQR* interquartile range, *CI* confidence interval

## Discussion

This pilot study established a yield of 6.6% new TB among household contacts where the TB index case 1) is a child < 5 years, 2) has HIV co-infected PTB (≥5 years), 3) has HIV-negative PTB (≥5 years) or 4) has MDR TB. A number of previous studies in sub-Saharan African countries examined the yield of TB among household contacts of different categories of TB index cases. Two sub-Saharan African country studies established a yield of 10% or higher new TB among household contacts – a Ugandan study targeting new sputum smear positive (SS+) index TB cases (≥15 years): 15.7% [26]; and an Ethiopian study of MDR TB index cases (all ages): 10.0% [27]. Other sub-Saharan African country studies produced a yield of between 5 and 8% – a study in North West Province of South Africa targeting newly diagnosed TB index cases (≥15 years): 7.8% [28]; a Kenyan study targeting SS+ TB cases (all ages): 6.7% [29]; an Ethiopian study targeting all types of TB cases (≥18 years): 6.5% [30]; and a Tanzanian study targeting laboratory-confirmed TB cases (all ages): 6.4% [12].

The 6.6% yield of new TB among the household contacts of the WHO-recommended infectious index case categories for SHCI observed in the current study, falls within the typical range of 5 to 8% observed in sub-Saharan African country studies utilising a targeted SHCI approach. Thereupon non-targeted household contact investigation studies in sub-Saharan African countries tend to demonstrate a lower yield. For example, a study in the North West Province of South Africa among adult TB index cases produced a yield of only 1.3% [31] and a study in Ghana among adult TB index cases returned a yield of only 0.65% [32].

The current study did not yield any new TB among household contacts of MDR TB index cases. A systematic review and meta-analysis [7] of the yield of active TB among household contacts of mono, poly, multi or extensively drug-resistant TB index cases recorded in 25 studies in 13 countries, including three studies in South Africa, showed that the yield varied from nil recorded in studies among adults in Taiwan [33] and Switzerland [34], to 23% recorded in a South African study of children evaluated as household contacts of adult MDR TB index cases in the Western Cape Province [35]. The lack of yield among contacts of MDR TB index cases in the current study could possibly be attributed to the fact that only one MDR TB patient contact was screened to be symptomatic and referred for clinical evaluation, subsequently yielding no TB.

Similar to findings of a study in India [36], and another in the North West Province of South Africa [28], the yield of new TB cases in the current study was highest among contacts of HIV-negative index cases. This finding could potentially be linked to the programmatic drive and recommendation for routine TB screening and evaluation of HIV-positive individuals, as they are particularly vulnerable and prone to the negative effects of late diagnosis and treatment of TB [6]; consequently, less attention may be given to HIV-negative TB patients. The finding of high prevalence of TB among household contacts of HIV-negative index cases underscores the need for TB programmes to direct ACF efforts, i.e., SHCI, beyond children < 5 years and HIV-positive cases, and to consider a broader range of risk categories.

Practical experience has shown that, in order to prevent laboratory overload, an average of seven (range: 3–20) people with presumptive TB should be screened to identify one SS+ case of TB [37]. In a resource-constrained country, such as South Africa, the NNS is particularly useful to determine the effectiveness of household contact screening within the context of resource scarcity [37, 38]. The NNS to diagnose one new TB case in the current study was 15, which lies within the range of 4–71 reported in a systematic review of contact tracing studies across Africa, Asia and the Americas [37]. The finding highlights the need to prioritise targeted SHCI as a strategy for ACF.

While there is widespread evidence that SHCI is effective, it is important that symptomatic and high-risk contacts present promptly at health facilities for clinical evaluation [5, 21]. In the current study, despite consistent efforts by the research team to follow up and encourage household contacts to attend clinical evaluation following non-compliance, less than half (47%) of those referred, underwent clinical evaluation. As reported by an earlier study in the Mangaung Metropolitan District [25], this failure to present was primarily due to individual (as opposed to health systems) reasons, such as lack of time, perceived inability to produce a sputum sample, lack of transport to clinics, and work or travel commitments. Similarly, in a Ugandan study, individual factors, including fear of stigma, limited knowledge, mistrust of health workers by both TB index cases and their contacts, and lack of time to travel to health facilities, emerged as key barriers to non-completion of clinical evaluation by contacts [21].

Ultimately, SHCI is about increasing early access to TB services. This pilot study suggests that alternative strategies by the TB programme in the Mangaung Metropolitan District, to ensure that symptomatic and high-risk contacts complete clinical evaluation, as well as efforts to ensure that they are linked to effective treatment and sustained care, may be necessary. In this study, efforts were made by fieldworkers to follow up and encourage those who had not undergone clinical evaluation, to do so. Under routine programme conditions, this necessitates dedicated human resources to conduct routine household screening coupled with continuous follow-up, to ensure clinical evaluation and treatment initiation. Previous research in South Africa [39] and Kenya [40] found that counselling by community health workers (CHWs) can have a positive impact on uptake of HIV counselling and testing services. A previous study among TB patients in the Free State established their satisfaction with and even preference for HIV counselling and testing delivery by CHWs instead of nurses [41].

The following limitations are associated with the current study. First, this was a pilot study, conducted over of a period of only 2 months, to gauge the effectiveness and yield of systematic screening and referral of household contacts for TB evaluation, in order to inform scale-up efforts in the Free State Province. Under ideal conditions, contacts can be visited and motivated more than twice, until they decide to undertake clinical evaluation. Second, due to purposive sampling of infectious index cases and their household contacts, the results are not generalisable to all contacts of TB patients in the province. Third, to allow for prompt referral, fieldwork was conducted within PHC facility operating hours. Some household contacts were missed, as the fieldwork teams were not able to conduct household visits during the evenings and over weekends. Fourth, due to lack of expertise and resources, the research team could not collect sputum samples from symptomatic contacts, and these contacts, therefore, had to be referred to PHC facilities. Similarly, asymptomatic contacts were not tested for HIV to determine their eligibility for Isoniazid preventive therapy (IPT). Thus, only children < 5 years were referred for clinical evaluation and/or IPT initiation at the PHC facilities.

## Conclusion

This pilot study shows that targeted SHCI using the WHO-recommended infectious TB index case categories can be effective. The results confirm the need to direct ACF efforts, such as SHCI beyond children < 5 years and HIV-positive cases, and to consider a broader range of risk categories. Results showed variation in the yield of TB across the different WHO-recommended TB index case categories, with the highest yield among contacts of HIV-negative PTB index cases, which are not currently prioritised. Routine SHCI of household contacts might improve the yield of ACF among HIV-negative TB and other high-risk infectious index cases and, particularly, as observed in this pilot study, male and coughing household contacts. For Mangaung and similar high-risk settings, this necessitates dedicated human resources towards routine SHCI, and concerted efforts to follow up contacts for clinical evaluation for TB.

## Data Availability

The data analysed during this study are not publicly available, as individual privacy could be compromised.

## Abbreviations

ACF: Active case finding
CHW: Community health worker
HIV: Human immunodeficiency virus
IPT: Isoniazid preventive therapy
MDR-TB: Multidrug-resistant tuberculosis
NNS: Number needed to screen
PHC: Primary health care
PTB: Pulmonary tuberculosis
SHCI: Systematic household contact investigation
SS+: Sputum smear-positive
TB: Tuberculosis
WHO: World Health Organization
